# Effect of Acupuncture on Gut-Brain Axis Parameters in Patients with Atopic Dermatitis: A Study Protocol for a Randomized, Participant- and Assessor-Blind, Sham-Controlled Trial

**DOI:** 10.1155/2021/5584247

**Published:** 2021-09-04

**Authors:** Jundong Kim, Soon-Kyeong Kwon, In-Seon Lee, Mijung Yeom, Dae-Hyun Hahm, Hi-Joon Park, Kyuseok Kim

**Affiliations:** ^1^Department of Ophthalmology, Otorhinolaryngology and Dermatology of Korean Medicine, Graduate School of Korean Medicine, Kyung Hee University, Seoul 02447, Republic of Korea; ^2^Division of Applied Life Science (Brain Korea 21 PLUS), Gyeongsang National University, Jinju 52828, Republic of Korea; ^3^Department of Korean Medical Science, Graduate School, Kyung Hee University, Seoul 02447, Republic of Korea; ^4^Acupuncture and Meridian Science Research Center, College of Korean Medicine, Kyung Hee University, Seoul 02447, Republic of Korea; ^5^Department of Physiology, College of Medicine, Kyung Hee University, Seoul 02447, Republic of Korea; ^6^Department of Ophthalmology, Otorhinolaryngology and Dermatology of Korean Medicine, College of Korean Medicine, Kyung Hee University, Seoul 02447, Republic of Korea

## Abstract

Atopic dermatitis (AD) is a relapsing and remitting chronic inflammatory skin disease for which a variety of etiological factors are involved. Treatment strategies should be multifaceted and have few side effects. In this respect, acupuncture has become increasingly popular as a safe, consistently effective, and drug-free therapy that treats multiple AD symptoms. We aim to not only verify the effectiveness of acupuncture but also suggest patient-specific response determinants and a new underlying mechanism implicating the gut-brain axis. We have designed a randomized, participant-blinded, sham-controlled clinical trial for 60 mild to moderate AD patients. In a previous study, we observed that the clinical skin symptoms of AD were closely associated with gastrointestinal (GI) symptoms. From these findings, we developed an intervention with six acupuncture points: three for AD symptoms and three for GI symptoms. Also, since high responders and low responders to the acupuncture treatment could be identified in the previous study, we now aim to explore response-determining factors, with a particular focus on GI symptoms. Therefore, we will precisely evaluate not only AD symptoms using the SCORAD, EASI, and DLQI tools, but also GI symptoms using the GSRS, TDS, BSFS, and AR tools and abdominal examination. AD develops in association with complicated pathophysiological factors, such as skin barrier function, genetic susceptibility, and immunological factors. Moreover, the underlying mechanism by which acupuncture treatment works has not been clearly elucidated. We, therefore, will conduct a simultaneous cross-sectional study with a sample of 40 healthy individuals, wherein potential indicators, such as fMRI, gut microbiota, and serum TARC and ATX, will be investigated to determine the gut-brain axis-associated mechanism of acupuncture. We expect that the results of this study could provide important clinical evidence for the effects of acupuncture and help elucidate the therapeutic mechanisms that underlie acupuncture's efficacy in AD treatment. This trial is registered with https://clinicaltrials.gov/ct2/show/KCT0005422 (Trial registration: Korean Clinical Trial Registry (http://cris.nih.go.kr; registration number: KCT0005422); date of registration: September 23, 2020).

## 1. Introduction

Atopic dermatitis (AD) is a relapsing and remitting, inflammatory, chronic skin disease that is characterized by severe itching and eczema [1]. Its prevalence varies widely according to geographic location, but according to an International Study of Asthma and Allergies in Childhood (ISAAC)-based report, it is on the rise worldwide, with a global prevalence of approximately 230 million [1, 2]. Additionally, the factors involved in AD pathophysiology are complex and diverse, and recent focus has mainly been on the hygiene hypothesis, loss-of-function mutations of the FLG-encoding gene, environmental factors, and the gut microbiota-skin axis [3].

However, AD cannot be cured [3]. Therefore, multistep guidelines have been outlined to control symptoms and maintain remission for as long as possible. However, in clinical practice, many patients do not respond properly to existing treatments. Persistent, recurring skin symptoms also exist because of treatment discontinuity. On the other hand, if continuous systemic treatment is administered, concerns about side effects and complications come to the forefront [4]. Therefore, to cope with the limitations of these existing treatments, the potential role of East Asian medicine, including Korean medicine, is being actively studied [5, 6]. A recent paper reported that acupuncture is a safe AD treatment that can be effective at improving itchiness, Eczema Area and Severity Index (EASI) scores, and other global symptoms [6].

Our research team has conducted previous randomized controlled trials (RCTs) addressing AD-related topics [7, 8]. Thirty-eight patients with AD were recruited, divided into two groups, and treated with verum or sham acupuncture twice a week for 4 weeks. In that study, acupuncture was performed on three fixed acupoints (ST36, LI11, and PC6 bilaterally) and two additional points. Additional points were selected on an individualized basis according to the accompanying symptoms, such as ST43 and GB41 for gastric stiffness and tenderness or LI2 and GB31 for diarrhea and constipation. Through this study, we not only confirmed the effectiveness of acupuncture treatment but also highlighted two notable implications. The first was that AD severity and the presence of gastrointestinal (GI) symptoms were positively correlated, and the second was that some patients exhibited clear improvements, but some patients did not.

Based on these two implications, the main purpose of this study is to explore the response determinants of acupuncture treatment. We plan to explore the underlying mechanisms simultaneously. Based on the gut-brain axis theory [9], we will analyze gut microbiota composition using fecal samples, and we will analyze brain activity and connectivity data using functional magnetic resonance imaging (fMRI). As links between fMRI and gut microbiota data, we will also collect serum thymus and activation-regulated chemokine (TARC) levels and autotaxin (ATX) levels.

## 2. Methods

### 2.1. Objectives

The primary purpose of the present study is (1) to determine the response determinants of acupuncture treatment and (2) explore the underlying mechanism of acupuncture, mainly focusing on the gut-brain axis.

### 2.2. Trial Design and Setting

**Figure 1:**
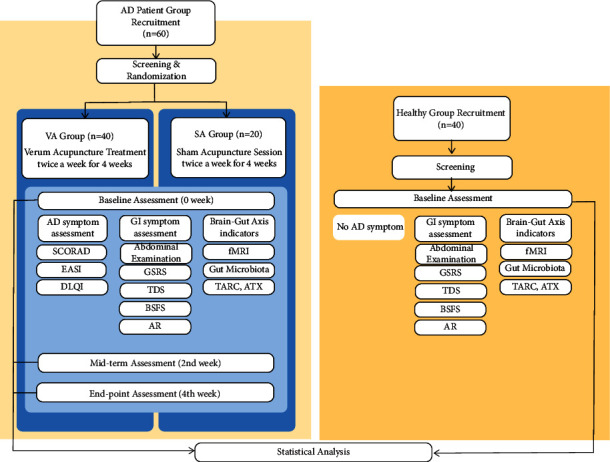
Study flowchart. The light-yellow area depicts the randomized controlled trial component, and the dark-yellow area depicts the cross-sectional component.

This study will combine an RCT and a cross-sectional component, as summarized by the study flowchart (Figure 1).

#### 2.2.1. Randomized Controlled Trial

The RCT component will include only 60 participants with mild to moderate AD. Forty and twenty participants will be randomly assigned to the verum acupuncture (VA) group and sham acupuncture (SA) groups, respectively. It will be a randomized, assessor- and participant-blinded, sham-controlled trial with a parallel-group design. All trial processes and procedures will be carried out at the Kyung Hee University Korean Medicine Hospital and Korea University Brain Imaging Center (KUBIC) in Seoul, Korea.

#### 2.2.2. Cross-Sectional Component

The cross-sectional component will include 40 healthy controls and 60 patients with AD. The study will be conducted at the Kyung Hee University Korean Medicine Hospital and KUBIC.

#### 2.2.3. Sample Size Calculation

This is not a confirmative study to evaluate the effectiveness of acupuncture. In our previous study [8], we have already confirmed the effectiveness of acupuncture treatment on patients with mild to moderate AD. The aim of this study is to discover the response determinants and the mechanism of action of acupuncture treatment for AD, mainly focusing on the gut-brain axis. Therefore, to calculate the sample size of this study, we considered a sample size of 18 per group calculated based on the SCORAD (total) score as the primary endpoint in our previous study [8] and a sample size of 12 in another study that had a similar design and explored treatment response determinants [10] as well as allocation of the verum acupuncture (VA) and sham acupuncture (SA) groups at a ratio of 2 : 1 to examine the difference in treatment response according to GI symptoms in the acupuncture treatment group. Considering the aim of the present study and the sample size of these previous studies, under the expert agreement, we calculated the required sample sizes for the AD VA, AD SA, and healthy control groups as 40, 20, and 40, respectively.

### 2.3. Ethics and Dissemination

The institutional review board (IRB) of Kyung Hee University Korean Medicine Hospital approved this study on July 21, 2020 (IRB No: KOMCIRB 2020-06-003). This study was registered in the Korean Clinical Trial Registry (CRIS, registration number: KCT0005422). All information regarding the study protocol will be provided to each participant. Written informed consent will also be obtained.

### 2.4. Eligibility Criteria

#### 2.4.1. Inclusion Criteria

*(1) Healthy Group*

1. Men and women aged 19 to 49 years able to read and write the Korean language
2. Body mass index (BMI) of 18.5 or more and less than 25 (kg/m^2^)
3. Right-handed
4. Do not have AD diagnosed in accordance with the Hanifin and Rajka criteria [11].
5. Agreement with the study protocol and providing written informed consent

*(2) AD Patient Group*

1. Men and women aged 19 to 49 years able to read and write the Korean language
2. Body mass index (BMI) of 18.5 or more and less than 25 (kg/m^2^)
3. Right-handed
4. AD diagnosed in accordance with the Hanifin and Rajka criteria [11].
5. Scores from 10 to 40 points of the objective Scoring Atopic Dermatitis (SCORAD) scale
6. Agreement with the study protocol and providing written informed consent
7. Do not participate in other clinical studies in the preceding month

#### 2.4.2. Exclusion Criteria

1. Those who have taken drugs (especially antibiotics), within 1 month before study participation that have the potential to affect the study results
2. Those who have taken functional foods for health (especially probiotics and prebiotics) within 1 month before study participation, which have the potential to affect the study results
3. Those who have a history of alcohol consumption within 1 week before study participation
4. Those with extensive tattoos or (semi-)permanent facial makeup
5. Those with claustrophobia
6. Those who cannot lie down for a certain amount of time due to pain or discomfort
7. Those with nonremovable implanted metal devices, such as pacemakers, clips, and implantations.
8. (For women) Those who are pregnant or breastfeeding
9. (For women) Those with intrauterine contraceptives
10. Those who have had eye surgery (excluding LASIK and LASEK)
11. Those who have been diagnosed with other diseases (neuropsychiatric disorders, brain surgery history, stroke, epilepsy, dementia, alcohol or drug use disorder, etc.) that may affect the results.
12. Those who have been diagnosed with somatosensory-related diseases (Raynaud's disease, chronic pain disease, etc.)
13. Smokers
14. Those who are adjudged to be unsuitable participants of this study based on physical or mental incompatibilities

### 2.5. Participant Recruitment

We will recruit 60 patients with AD and 40 healthy controls. Recruitment will mainly proceed via bulletin board posts in the hospital. We plan to also postannouncements in nearby town offices and campuses.

### 2.6. Randomization and Allocation Concealment

Participants who meet the criteria and sign a consent form will be randomly assigned. Block randomization will be conducted to form the verum acupuncture (VA) and sham acupuncture (SA) groups at a ratio of 2 : 1, and the specific randomization method will proceed as follows.

An independent statistical expert will generate a random number table using random allocation software (SAS 9.2 (PROC PLAN, SAS Institute Inc., Cary, NC, USA)). This random number table will be sent to administrative staff at the Acupuncture and Meridian Science Research Center (AMSRC), who are not involved in assessing or recruiting the participants. The staff will place each random number in a sealed, opaque envelope and store them. After the screening, the investigator will contact the administrative staff by e-mail or phone, and the personnel will immediately forward a document that contains the group allocation and participant's unique random number. The investigator will keep this e-mail in the trial master file in printed form.

There will be no randomization of the 40 healthy controls. Only 25 of them can volunteer for fMRI Task 2, which will be conducted on a first-come, first-served basis.

### 2.7. Blinding and Code-Breaking

In the AD group, the participants will be blinded. During the acupuncture sessions, a screen will be erected so that the participants cannot see the Korean medical doctor (KMD) who applies the acupuncture treatment. However, due to the characteristics of acupuncture intervention, it is impossible to blind the acupuncture practitioner. However, the KMD administering acupuncture will not participate in data collection, analysis, and evaluation of results; these functions will be applied by blinded investigators. If serious adverse reactions occur or the participant asks to pause or terminate the session, the acupuncture treatment will be immediately stopped, and the blinding code will be broken.

### 2.8. Intervention

The intervention schedules for each group are shown in Tables 1 and 2.

### 2.9. Acupuncture Procedure

This study will be conducted based on the Revised Standards for Reporting Interventions in Clinical Trials of Acupuncture (STRICTA) 2010 checklist (Table 3) [12]. Acupuncture will be performed by a KMD who had graduated from a college of Korean medicine with a 6-year course and had been certified by the Korean Ministry of Health and Welfare. With at least 2 years of clinical experience, a KMD who performs all the acupuncture sessions will perform more than 10 hours of training and prior practice. This is a procedure to provide standardized acupuncture sessions consistently according to the prescribed manual of the standard operation procedure (SOP).

*(1) Verum Acupuncture (VA) Group*. The VA session will be conducted twice a week for 4 weeks, and the visit window period will be ±5 days relative to the scheduled date. Participants in the VA group will undergo acupuncture at six acupoints. PC6, LI11, and ST36 will be treated on both sides. GB41, ST43, and LI3 will be treated on only one side—the left side for men and the right side for women. In principle, acupuncture needles will be inserted vertically into all acupoints, and the insertion depth will range from 5 mm to 30 mm, depending on the acupoint location. Acupuncture needles will remain in place for 15 minutes. All acupuncture needles used for participants in the VA group will be disposable sterile stainless steel needles (0.25 × 40 mm; Dongbang Acupuncture Inc., Bundang, Seongnam, Korea). All acupuncture treatments will start after the participants take a 5-minute break upon their arrival.

*(2) Sham Acupuncture (SA) Group*. Sham acupuncture will be conducted twice a week for 4 weeks, and the visit window period will be ±5 days relative to the scheduled date. The SA body sites and depths of acupuncture will differ from those in the VA group. The SA locations will be as follows:

- 1–2 cm proximal to and 1 cm in the ulnar direction away from PC6 on both sides
- 1 cm proximal to and 1 cm in the ulnar direction away from LI11 on both sides
- 1 cm proximal to and 1 cm in the fibular direction away from ST36 on both sides.

There is no depth standard for SA because SA only involves skin stimulation and not penetration. Sham needles will remain in place for 15 minutes. Park Sham Needles and nonpenetrating sham devices (Park Sham Acupuncture Needles, AcuPrime Co., Ltd., Exeter, UK) will be used for all SA sessions. All sessions will start after the participants take a 5-minute break upon their arrival.

### 2.10. Compliance and Discontinuation

Based on our previous pilot study [8], we concluded that if the study participants receive six to eight acupuncture sessions in compliance with the regulations, this will be sufficient to generate valid and reliable data. For participants who drop out before their sixth acupuncture session, data from the last visit will be used for analysis. The research coordinator will regularly encourage participants to continue to the end of the trial.

### 2.11. Outcome Measures

The study outcomes will be evaluated by an investigator blinded to the treatment assignments. There will be no separation between primary and secondary outcomes since the aim of this study is not to confirm the effectiveness of acupuncture treatment in patients with AD but to discover the response determinants and the mechanism of action of acupuncture treatment for AD, mainly focusing on the gut-brain axis.

Clinical outcomes can be divided into three categories: First, we will assess (1) symptoms of AD (changes in SCARAD, EASI, and Dermatology Life Quality Index [DLQI] assessments). We will also assess (2) GI symptoms (changes in abdominal examination findings, as well as changes in scores according to the Gastrointestinal Symptom Rating Scale (GSRS), Total Dyspepsia Symptom (TDS) scale, Bristol Stool Form Scale (BSFS), and the adequate relief of functional dyspepsia pain and discomfort (AR) assessment). These values will be measured at week 0 (baseline), week 2 (midterm), and week 4 (endpoint).

Finally, since this study aims to explore the mechanism of acupuncture, we will also investigate (3) gut-brain axis indicators. First, the changes in the intensity of blood-oxygen level-dependent (BOLD) signals in the pain and itch matrix, including the thalamus, insula, and cingulate cortex, will be measured using fMRI. Additionally, the functional connectivity between the pain and itch matrix regions will be analyzed. Second, a fecal sample will be taken, and the composition of the gut microbiota in the feces will be analyzed. Blood samples will also be collected and analyzed for TARC and ATX in the serum.

#### 2.11.1. AD Symptom Assessments

*(1) Scoring Atopic Dermatitis (SCORAD)*. The SCORAD score was developed by the European Task Force on Atopic Dermatitis (ETFAD) as a tool to assess the severity of AD [13]. It evaluates not only objective signs but also subjective symptoms, such as itching and insomnia.

*(2) Eczema Area and Severity Index (EASI)*. The EASI is considered a complementary evaluation tool to the SCORAD scale because it more specifically evaluates symptoms of acute and chronic inflammation [14].

*(3) Dermatology Life Quality Index (DLQI)*. The DLQI is a tool for evaluating the quality of life, and it can evaluate psychological distress caused by skin symptoms [15]

#### 2.11.2. GI Symptom Assessments

*(1) Abdominal Examination*. Abdominal examination is a commonly used diagnostic method in Korean medicine. Abdominal examination in Korean medicine differs from the physical abdominal examination of Western medicine because the Korean variation follows the Korean medical theory that specific responses appear at the body surface (abdomen) when the disease exists inside the human body. Therefore, in this study, we will use abdominal examination to detect GI symptoms [16]. Specific details include evaluation of tenderness in CV12 and ST25 on both sides and confirmation of the presence of stuffiness below the heart (*Simhabi, 心下痞*) and stuffiness and rigidity below the heart (*Simhabikyung,* 心下痞硬). Both *Simhabi* and *Simhabikyung* are symptoms that occur in the anticardium (the area encompassing CV13 and CV14). The former, *Simhabi*, includes the patient's feeling of fullness, bloating, stuffiness, and blockage in the epigastric region, which is felt only subjectively, but there is no objective resistance or tenderness. It includes all kinds of stomach upset, epigastric fullness after meals or as usual. *Simhabikyung* refers to a case in which a doctor can sense resistance in the epigastric region objectively by hand [17, 18].

*(2) Gastrointestinal Symptom Rating Scale (GSRS)*. The GSRS can be used to evaluate both upper and lower GI symptoms. In addition to pain, soreness, and indigestion affecting the upper GI tract, the characteristics of the stool and discomfort can also be evaluated together [19].

*(3) Total Dyspepsia Symptom Scale (TDS)*. The TDS is a tool used primarily for evaluating symptoms in patients with functional dyspepsia, particularly postprandial bloating, premature satiety, nausea, vomiting, and belching [20].

*(4) Bristol Stool Form Scale (BSFS)*. The BSFS is a proper tool in daily clinical practice. It is a quick, easy, and intuitive patient-reported measure of whole-gut transit time, and it can be used as a substitute for stool consistency [21].

*(5) Adequate Relief of Functional Dyspepsia Pain and Discomfort (AR)*. The content is as follows: “Have you experienced appropriate relief (more than 50% reduction) of the pain or discomfort associated with your dyspepsia after the last acupuncture treatment?” AR asks about the degree of improvement in general and nonspecific digestive discomfort [7].

#### 2.11.3. Gut-Brain Axis Indicators

*(1) Fecal Sampling and Microbiota Analysis Protocol*. For each participant, approximately 200 mg of fecal samples will be employed as an input for DNA extraction with the FastDNA for the fecal kit (MP Bio, Santa Anna, CA, USA) following the manufacturer's instructions. The V3-V4 hypervariable regions of the 16S rRNA gene will be targeted using a universal primer set (5ʹ-CCTACGGGNGGCWGCAG and 5ʹ-GACTACHVGGGTATCTAATCC) with sequencing barcodes. Sequencing will be performed on an Illumina MiSeq platform with 2 × 250 bp paired-end protocol yielding pair-end reads. We mainly follow the QIIME2 pipeline [8] for bacteria profiling based on 16S rRNA. DADA2 will be selected as a tool for sequence quality control, and the EzTaxon [9] database will be chosen as the taxonomic reference database. The results will be imported into the *R* statistical environment (The *R* Foundation, https://cran.r-project.org/) for further analysis using the Bioconductor package phyloseq [10]. Alpha-diversity will be determined by the value of clustered observed operation taxonomic units (OTUs), Shannon index, and the inverse Simpson index. Beta diversity will be assessed using weighted or unweighted UniFrac distance matrices.

*(2) Blood Sampling and Analysis of TARC and ATX*. Blood samples will be collected in the blood collection room on the second floor of Kyung Hee Medical Center. After clotting, the serum will be separated by centrifugation at 3000 rpm for 10 minutes and stored at −80°C. Analysis of TARC and ATX will be conducted using commercial ELISA kits at the AMSRC.

*(3) fMRI*. (i) Preevaluation for fMRI sessions: interoceptive and exteroceptive properties for each individual will be measured before the fMRI sessions; (ii) fMRI session: (a) fMRI data acquisition—fMRI data on a 3 T MRI scanner (Siemens, Erlangen, Germany) with a three-axis gradient head coil will be acquired. Anatomical images using a T1-weighted rapid gradient-echo sequence with TR = 2000 ms, TE = 2.37 ms, a flip angle of 9°, a field of view of 240 × 240 mm^2^, and a slice thickness of 1.0 mm will be acquired. Resting-state and task-related functional images will be acquired with a T2∗-weighted gradient-echoplanar imaging sequence (37 slices; TR = 2000 ms; TE = 30 ms; flip angle, 90°; field of view, 240 × 240 mm^2^; slice thickness, 4.0 mm; voxel size = 3.8 mm 3.8 mm × 4 mm^3^). (b) Video watching and sensory imagination tasks—the stimulus materials consist of various types of videos, which were recorded by the researchers, belonging to four categories: images showing painful events (pain condition; acupuncture needle rotation, hammer hitting fingers, knife cutting skin, pinprick pricking face, etc.), images evoking itch (itch condition; scratching neck, arm, leg, chest, and abdomen), images showing nonpainful events (control condition for pain condition; depictions of cotton, pen, straw, brush on hand, arm, and face), and images evoking nonitch rest (control condition for itch condition; tapping neck, arm, leg, chest, and abdomen). Pain and nonpain videos, itch and nonitch videos were matched each other in terms of velocity of the motion, body part, model, background, and context. Items for pain, nonpain, itch, and nonitch videos were used in previous pain imaginary task studies [22] and contagious itch studies [23]. Itch and nonitch videos will depict female or male researchers performing scratching or tapping behavior without revealing their faces, female participants will watch videos recorded with female researchers, and male participants will watch videos recorded with male researchers. Participants will be asked to imagine that the stimuli (painful or nonpainful) and behaviors (scratching or tapping) shown in the videos are happening to them while watching the videos. Each video will continue for 2 seconds, and each trial will consist of four consequent videos of the same category. The order of trials will be pseudorandomized without repetition of two consecutive categories. In each block, 24 trials (six trials per condition) will be presented with a randomly generated interstimulus interval (minimum interval, 10 seconds; maximum interval, 20 seconds; mean of intervals, 15 seconds). All participants will complete an fMRI session consisting of five blocks, and they will have as much rest as they request between each block. After each block, participants will rate verbally how much they experience itch and pain sensations while watching the videos (0: not painful/itchy at all, 10: very much painful/itchy). In total, the duration of the fMRI scan will be approximately 60 minutes for all participants.

### 2.12. Statistical Analysis

The data analysis will be performed by the statisticians independent from the research team, and the blinding will be maintained. The statisticians will be experts affiliated with the Kyung Hee University Medical Research Institute.

#### 2.12.1. Statistical Analysis for AD Symptom and GI Symptom Assessments

The continuous variables, including SCORAD, EASI, DLQI, GSRS, and TDS scores, will be presented as mean ± standard deviations with 95% confidence intervals. In the analysis, if the result values follow the mean distribution, the independent two-sample *t-*test can be used; otherwise, the Mann–Whitney *U* test can be used.

On the other hand, the abdominal examination, BSFS, and AR variables are categorical. These will be expressed as *n* (%), and as mentioned above, these will be analyzed using the chi-square test or Fisher's exact test. Data from the 40 participants in the VA group will be compared with those of the 20 participants in the SA group.

For the 40 participants in the VA group, additional analyses will be conducted. With the primary endpoint being the total SCORAD score, we will first analyze the correlation as a continuous variable through regression analysis. The dependent variables will include GSRS, TDS, BSFS, and AR values, as well as abdominal examination findings, which are indicators of GI symptoms.

In addition, based on the above data, we will explore the cutoff total SCORAD value that can distinguish between high and low responders. The reference value set through this process will be divided into categorical variables to evaluate the association with GI symptoms again. The minimal clinically important difference (MCID) acts as an important cutoff that determines whether a response is clinically useful, meaning 8.7 points in total SCORAD and 8.2 points in objective SCORAD [24]. The MCID value will be used for analysis as a reference cutoff value. The purpose of this study is to identify factors associated with response determination by performing a correlation analysis with the above method.

#### 2.12.2. Statistical Analysis for Gut-Brain Axis Indicators

Changes in the gut-brain axis indicators before and after acupuncture will be analyzed within and between groups.

*(1) Gut Microbiota Analysis*. The significance of categorical variables will be determined using the nonparametric Mann–Whitney test for two category comparisons or the Kruskal–Wallis test when comparing three or more categories. Multivariate analyses of variance (PERMANOVA, 999 permutations) with the vegan function Adonis will be performed to test whether community composition is significantly different according to the features associated with sequencing data.

*(2) Serum TARC, ATX Analyzing*. The significance of continuous variables will be determined using the independent two-sample *t*-test or the Mann–Whitney *U* test. The variables will be presented as mean ± standard deviation with 95% confidence intervals.

*(3) fMRI Analysis*. (i) Preevaluation for fMRI session: the between-group and AD severity-dependent differences in interoceptive and exteroceptive properties will be analyzed using mixed-model linear regression and analysis of variance (ANOVA), with the subject as a random factor. Interoceptive and exteroceptive outcomes will be correlated with functional brain activities (voxel-wise beta-series from sensory imagination tasks) and subjective itch and pain ratings of the tasks, which will be performed during fMRI scanning using Pearson's correlation analyses with and without covariates (AD severity, group). If nonparametric data in one or both variables were present, Spearman's rank order correlation will be used. (ii) fMRI data: raw fMRI data will be preprocessed with Analysis of Functional NeuroImages (AFNI, https://afni.nimh.nih.gov) including slice time correction, alignment, registration, masking, smoothing using a Gaussian kernel, and scaling. For the resting-state fMRI signals, the seed-based amplitude of low-frequency fluctuations, functional connectivity, and graph theory analyses will be performed. Task-related BOLD signals will be compared as a function of the four different types of imagination tasks described below using general linear model tests.

Additionally, trial-specific estimates for each task will be obtained using beta-series regression and a least-squares model, and then the resulting parameter estimates will be used as input features for multivariate pattern analysis. (iii) Video watching and sensory imagination tasks: all intensity ratings will be correlated with functional brain activity and symptom-related questionnaire scores. The effects of sensory imagination tasks and between-group differences, as well as their interactions on subjective ratings, will be analyzed using mixed-model linear regression and ANOVA. Mediation analysis will be performed to identify the factors that mediate brain responses during sensory imagination tasks.

*(4) Multivariate Analysis of Gut-Brain Axis*. For the multivariate analysis of microbiome data, potential microbiome-phenotype associations will be quantitatively assessed by classification and regression models using random forest (RF) and support vector machine (SVM). On microbial sequencing data, relative abundances of operational taxonomic units (OTUs) or collapsed OTUs into genus or species will be used for the microbiome features. For the multivariate analysis of fMRI time series, trial-specific estimates for each task will be obtained using beta-series regression and a least-squares model, and then the resulting parameter estimates will be used as input features for multivariate pattern analysis. The most important discriminating features and their importance scores will be retrieved by running RF and SVM models for AD discrimination (group identification, prediction of disease severity, etc.). The performances of RF and SVM models will be statistically evaluated using mean-square error, coefficient, accuracy, and area under the curve to find the best prediction model and its parameters.

Multicollinearity will be with variance inflation factor (VIF), which identifies the strengths of the correlations between independent variables. We will either exclude variables with high VIF values from the regression analysis or use high VIF values if they contribute to the model.

### 2.13. Participant Safety

All participants will be fully informed about the study protocol prior to participation. They will be asked to provide informed consent in writing. This process will be conducted at the very beginning of the trial process. Participants will be informed about and monitored for any potential adverse events of acupuncture treatment at each visit. Adverse events will be recorded on a worksheet and case report form. If a serious adverse event occurs, the case will be immediately reported to the clinical trial director and the IRB at Kyung Hee University Korean Medicine Hospital. If necessary, part of the trial will be halted until further instructions are available.

### 2.14. Confidentiality

All documents will be categorized by unique codes but not by participants' names to protect confidentiality. All participant identification information will be kept confidential and will only be viewable by researchers. All data and records will be securely locked under the administration's protection for three years after the publication of the trial results and then destroyed. The human-derived fecal and blood samples will be stored for as long as the participant agrees (Two years will be recommended).

### 2.15. Quality Control

All study researchers will receive centralized and unified training before the study and all the processes will be conducted under the SOP. This study will be performed under the monitoring of the Korean Medicine Clinical Trial Center at Kyung Hee University Korean Medicine Hospital and the AMSRC. If a protocol modification is required due to unanticipated problems, representatives of this study will discuss the matter fully for the appropriate steps.

## 3. Discussion

This will be a randomized clinical, practical, sham-controlled trial with participant and assessor blinding for AD patients. The RCT will include VA or SA sessions twice a week for 4 weeks. This treatment dose was determined based on our previous studies [7, 8].

AD patients participating in this RCT will be recruited if they have an objective SCORAD score of 10 to 40 points. The lower limit was set at 10 points because there must be enough skin symptoms to evaluate the effectiveness of acupuncture treatment. On the other hand, patients with severe AD will be excluded to allow them to continue or seek AD treatments other than acupuncture (which will be prohibited for study participants). These SCORAD score cutoffs are the same as the standards used in our previous studies [7, 8].

The acupoints will be PC6, LI11, and ST36, which are expected to improve skin symptoms, and ST43, GB41, and LI3, which are expected to act on GI symptoms [25–27]. The former three acupoints, PC6, LI11, and ST36, have been confirmed to improve skin symptoms in patients with AD in two previous studies [7, 28]. PC6 is not only an acupoint that can be used symptomatically for skin itching but is also known to have a positive effect on the stabilization of autonomic nerve function [29]. In addition, LI11 was found to improve itching and lichenification in patients with AD [30, 31]. ST36 is effective in regulating itching in various dermatitis diseases, including atopic dermatitis, and its representative mechanisms include negative feedback action on IL-33 and inhibition of mast cell degranulation [32–35]. In particular, the latter three acupoints (ST43, GB41, and LI3) were selected based on the Korean traditional acupuncture method, the Saam acupuncture [26]. The combination of ST43-GB41 is widely used in the clinical symptoms of the GI tract, especially in the upper GI tract. The combination of GB41-LI3 is used for lower GI symptoms [27]. In summary, the acupoints of our study are a treatment that can improve not only skin symptoms but also overall GI symptoms.

The main purpose of this study is to investigate response determinants of acupuncture treatment. We hypothesized that the presence of GI symptoms may be a strong contributory factor. Various hypotheses have been proposed to explain the basis of the acupuncture mechanism of action and responses to treatment, but particular attention has been paid to Th2 hypersensitivity reactions in the GI tract mucosa [36]. In people with genetic susceptibility to AD, when the Th2 reaction in the duodenal mucosa is activated by a specific antigen, eosinophil degranulation is induced by IL-5, and various other cytokines are released, resulting in neuronal action potentials. Consequently, muscle contractions and pain are induced in the GI tract, and these factors delay gastric emptying. In association with AD, it has also been suggested that Th2 hypersensitivity reactions represent pathophysiology. Thus, we hypothesized that this hypothesis is an appropriate basis for the correlation between the severity of AD and GI symptoms. For 60 AD patients, subgroups will be divided according to the presence or absence of GI symptoms so that the degree of improvement in each group will be compared. With this analysis, the possible significance of AD patients with GI symptoms being more sensitive to acupuncture can be assessed statistically.

This study will focus on the activity of the gut-brain axis. Accumulating evidence suggests that the optimal diversity of the gut microbiota is essential not only for the health of the gut but also for the general physiological functions of other organs, especially the brain. The communication pathways between the intestine and brain include the vagus nerve and spinal pathway, short-chain fatty acids (butyrate, propionate, and acetate), various cytokines (IL-1 and IL-6), and tryptophan secreted from immune cells and various neurotransmitters. The hypothesis that communication is carried out through the hypothalamus-pituitary-adrenal axis, which is the key to the regulation of stress, has also been proposed [37]. Moreover, the relationship between the gut-brain axis and skin is being studied in the context of research on various skin diseases, such as psoriasis, acne vulgaris, rosacea, and eczema [25, 26]. The mechanism by which the gut-brain axis is associated with the skin has emerged around the inhibitory effects of neurogenic skin inflammation according to stress regulation [38].

Therefore, in this study, we will confirm the activity of each region and the linkage through fMRI as the main indicator of the gut-brain axis. The difference in brain activity between patients with AD and healthy individuals is actively studied [28, 29], and our study will also investigate changes in activity before and after acupuncture. Additionally, the diversity of the gut microbiota and the dominant strain in AD patients have been discussed [39, 40]. In our study, the gut microbiota of the VA treatment group, the SA treatment group, and healthy controls will be compared. Moreover, as mediators connecting the brain and gut, hematologic indicators, TARC and ATX, will be measured together. Effector/memory CD4+ T cells play an important role in allergic diseases. TARC is produced in the intestinal endothelium and epithelial keratinocytes and is known to play a key role in the development and activation of Th2-type inflammatory mediators as well as inducing T-cell chemotaxis [41]. ATX is known to exhibit high mRNA activity in the brain, adipose tissue, ovaries, and small intestine [42], and it has been shown to cause pruritogenic-mediating activity through an ATX-lysophosphatidic acid signaling process in animal studies [43]. Based on these observations and facts, TARC and ATX will be evaluated as mediators that connect the brain, intestine, and skin. The collection and evaluation of these multifaceted data is an advantage of this study.

## Figures and Tables

**Table 1:** Study schedule for the AD patient group.

Outcome values and intervention		Baseline assessment	Treatment period								Final assessment
			1-week		2-week		3-week		4-week		
AD symptoms	SCORAD assessment	●				●					●
	EASI assessment	●				●					●
	DLQI assessment	●				●					●

GI symptoms	Abdominal examination	●				●					●
	GSRS assessment	●				●					●
	TDS assessment	●				●					●
	Bristol stool scale	●				●					●
	AR assessment			●	●	●	●	●	●	●	●

Gut-brain axis	BDI STAI-X1, 2	●									
	fMRI image (tasks 1 and 3)	●									●
	Stool collection	●									●
	Blood collection	●									●

	Acupuncture treatment		●	●	●	●	●	●	●	●	

	Investigate for adverse events		●	●	●	●	●	●	●	●	●

BDI, Beck Depression Inventory; STAI, State-Trait Anxiety Inventory; SCORAD, Scoring Atopic Dermatitis; VAS, Visual Analog Scale; EASI, Eczema Area and Severity Index; DLQI, Dermatology Life Quality Index; GSRS, Gastrointestinal Symptom Rating Scale; TDS, Total Dyspepsia Symptom Scale; AR, adequate relief of functional dyspepsia pain and discomfort; AD, atopic dermatitis. Except for the fMRI session, the baseline assessment will take between 30 minutes and 1 hour. An fMRI session, performed at the first and last evaluations, will take about an hour and a half to two hours. Each acupuncture session will take about 15 to 20 minutes. And a total of 30 to 45 minutes will be required for the questionnaires administered in the intermediate and final evaluations. Visit window is ±5 days. Baseline assessment—first acupuncture treatment and eighth acupuncture treatment—Endpoint assessment will be performed within 7 days. The visit window period is ±5 days.

**Table 2:** Study schedule for the healthy controls.

	Measurements	Assessment	Optional assessment (*n* = 25)
Gut-brain axis	BDI STAI-X1,2	●	
	fMRI image (tasks 1 and 3)	●	
	fMRI image (task 2, *n* = 25)		●
	Stool collection	●	
	Blood collection	●	

BDI, Beck Depression Inventory; STAI, State-Trait Anxiety Inventory. Except for the fMRI session, the baseline assessment will take between 30 minutes and 1 hour. An fMRI session will take about an hour and a half to two hours.

**Table 3:** Revised STRICTA checklist.

Item	Detail
(1) Acupuncture rationale	(1a) Style of acupuncture (i) Manual acupuncture (1b) Reasoning for treatment provided, based on historical context, literature sources, and/or consensus methods, with references where appropriate (i) Through a previous study conducted by this research team, it was confirmed that AD patients could be treated with acupuncture twice a week for 4 weeks to improve symptoms. In addition, the Acupuncture and Meridian Science Research Center textbook was referenced. Optimal acupoints were selected through clinical experience and consensus by the experts in acupuncture and AD optimal acupoints. (1c) Extent to which treatment was varied (i) No other interventions other than acupuncture.

(2) Details of needling	(2a) Number of needle insertions per subject per session (mean and range where relevant) (2b) Names (or location if no standard name) of points used (uni/bilateral) (i) Fixed points: 6 acupoints with 9 needles per participant per session: (ii) PC6, LI11, ST36 bilaterally, ST43, GB41, LI3 contralaterally (2c) Depth of insertion, based on a specified unit of measurement or on a particular tissue level from 5 to 30 mm, perpendicular to skin surface (2d) response sought (e.g., de qi or muscle twitch response) “De qi” sensation (2e) Needle stimulation (e.g., manual, electrical), manual stimulation, needle rotation with thumb and index fingers for the first 10–15 seconds No other stimulation sensations. (2f) Needle retention time 15 minutes (2g) Needle type (diameter, length, and manufacturer or material) A sterilized stainless steel needle (0.25 × 40 mm, Dong Bang Acupuncture Inc., Bundang, Seongnam, Korea)

(3) Treatment regimen	(3a) Number of treatment sessions (i) 8 sessions (3b) Frequency and duration of treatment sessions (i) Twice a week for 4 weeks, 15 minutes for each session (ii) Visit window: ± 5 days

(4) Other components of treatment	(4a) Details of other interventions administered to the acupuncture group (e.g., moxibustion, cupping, herbs, exercises, lifestyle advice) (i) Lifestyle advice will be given to all participants (ii) Any other interventions will be prohibited during the study period (4b) Settings and context of treatment, including instructions to practitioner and information and explanations to patients (i) Participants will be informed about acupuncture treatment in the study as follows: “In this study, you will be randomly allocated to the VA group or SA group. Acupoints will be selected as guided by traditional Korean medicine textbooks and AD-related reports.”

(5) Practitioner background	(5) Description of participating acupuncturists (qualification or professional affiliation, years in acupuncture practice, other relevant experience) (i) Acupuncture treatment in this study will be performed by a KMD who is majoring under the guidance of a Korean medical dermatologist. The clinical experience of this KMD is more than 2 years. The practitioner KMD will have to undergone more than 10 hours of training and simulation workshopping to ensure that he is able to provide consistent acupuncture treatment in accordance with a predefined protocol.

(6) Control and comparator interventions	(6a) Rationale for the control or comparator in the context of the research question, with sources that justify this choice (i) A control group (SA group) will be treated with sham acupuncture (SA) using park sham acupuncture Needles. (6b) Precise description of the control or comparator. If sham acupuncture or any other type of acupuncture-like control is used, provide details as for items (1) to (3) above (ii) Park sham acupuncture needles and devices will be used in the same environment as in the VA group. However, acupoints will be different: a Point 1 to 2 cm proximal and 1 cm medial to PC6, a point 1 cm proximal and 1 cm medial to LI11, and a point 1 cm proximal and 1 cm lateral to ST36, each bilaterally.

STRICTA: Standards for Reporting Interventions in Clinical Trials of Acupuncture; AD: atopic dermatitis; VA: verum acupuncture; SA: sham acupuncture; KMD: Korean medicine doctor.

## Data Availability

No data were used to support this study.
